# Risk-based prioritization of official animal welfare inspections using machine learning

**DOI:** 10.3389/fvets.2026.1938469

**Published:** 2026-09-30

**Authors:** Eva Obenaus, Sabrina Kuchling, Tadej Zerak, Corina Schleicher, Ian Kopacka, Sven Budik, Sabine Grimme, Jörg Hiesel, Peter Eckhardt, Johannes Baumgartner, Clair L. Firth

**Affiliations:** 1 Centre for Veterinary Public Health and One Health, Clinical Department for Farm Animals and Food System Transformation, University of Veterinary Medicine Vienna, Vienna, Austria; 2 Department of Veterinary Administration, Styrian Provincial Government, Graz, Austria; 3 Division of Data, Statistics and Risk Assessment, Austrian Agency for Health and Food Safety (AGES), Graz, Austria; 4 Animal Welfare Science Unit, Clinical Department for Farm Animals and Food System Transformation, University of Veterinary Medicine Vienna, Vienna, Austria

**Keywords:** animal welfare, animal welfare violations, data-based indicators, machine learning model, risk-based sampling, welfare violation indicator

## Abstract

**Introduction:**

For official animal welfare inspections in Austria, at least 2% of agricultural livestock farms must be selected and inspected on the basis of a risk-based random sample. In the federal state of Styria, more than 19,000 farms keep livestock, while the inspection capacity of official veterinarians is limited, requiring efficient prioritization of farm checks. This study aimed to develop a machine learning model leveraging routinely collected administrative and animal health data to identify farms in Styria at higher risk of animal welfare violations.

**Methods:**

The statistical analyses incorporated the outcomes of animal welfare inspections from 2019 to 2023, aggregated by farm and year. A total of 2,647 aggregated inspection results were used to train an Extreme Gradient Boosting (XGBoost) model, with a further 475 results used for testing purposes. Information about farms (e.g., production type, farmer and regional data, livestock data), as well as slaughter and meat inspection data, was acquired from the Consumer Health Information System database (*Verbrauchergesundheitsinformationssystem*, VIS). Additionally, data from local fallen stock rendering plants and information on antibiotics dispensed to farmers were available and incorporated into the model.

**Results:**

Results showed a moderate model performance with AUC (Area under the curve) of 0.75 (95%-CI [0.66, 0.82]), MCC (Matthews Correlation Coefficient) of 0.25 (95%-CI [0.19, 0.37]) and AP (Average Precision) of 0.19 (95%-CI [0.11–0.31]) for the test set. *Post-hoc* model interpretation revealed as most important influencing features antibiotic dispensing in cattle production, particularly overall quantities and European Medicines Agency Category D antibiotics, followed by farmer-related characteristics such as farmer age and the distance between the farm and the farmer’s residence. Membership in the Animal Health Service (*Tiergesundheitsdienst*, TGD) was associated with a decrease in the model-predicted probability of animal welfare violations. Other relevant factors included the number of livestock units (cattle and poultry) on farm, cattle mortality recorded by rendering plants, and certain findings at meat inspection, such as pericarditis in pigs.

**Discussion:**

These results indicate that routinely recorded administrative and veterinary data can provide meaningful indicators for identifying farms with a higher risk of animal welfare violations. Although some predictors appear indirectly linked to welfare, they likely reflect broader farm management practices and animal health status. Risk-based sampling could support veterinary authorities in prioritizing farms for inspection and allocating inspection resources more efficiently.

## 1 Introduction

According to the Austrian Animal Welfare Inspection Ordinance, 2% of agricultural livestock farms in Austria must undergo an official animal welfare inspection each year. Farms selected for inspection must be chosen on the basis of risk (1). In the federal state of Styria, 19,549 farms were reported to keep livestock in 2023 (2). The 2023 Austrian Agricultural Structure Survey, reported average herd sizes of 36 head of cattle, 469 chickens, 119 pigs, 35 sheep and 14 goats per farm, with Styria accounting for 26.7% of the total pig and 32.4% of the total chicken population in the country (3). By contrast, only 38 official veterinarians were responsible for animal welfare inspections in Styria in 2021 (4). A study from Finland showed that animal welfare checks are a particularly stressful part of official veterinarians’ work (5). To carry out the required number of annual welfare inspections, the official veterinarians in Styria have to inspect around 400 farms per year. In recent years, scientific research has increasingly focused on identifying indicators that can be used to predict animal welfare violations (6–10). Various studies have attempted to address this issue using data-driven and/or animal-based analytics (7, 9, 10). Private data sources, such as monitoring biometric parameters or performance data, could be an efficient method of observing animal health and welfare and it would be highly interesting to use these data for violation prediction (11). However, although these parameters could be highly beneficial for animal welfare predictions, these data are often owned by the farmers and/or private organizations and are therefore not available to public veterinary authorities for analysis (9). Investment in monitoring systems is often associated with an increased focus on animal welfare and fewer violations, which should be encouraged and promoted, and the possibility that such systems could be used for welfare surveillance by national authorities may lead to reduced uptake among farmers. Nevertheless, livestock farms generate a large amount of data, some of which are collected to comply with regulatory requirements or to meet the requirements of various agri-marketing programs and are accessible to official authorities.

Risk-based inspection systems generally comprise two related components: the estimation or categorization of risk and the subsequent allocation of inspection resources. Risk may be estimated using expert-based assessments, predefined risk scores, scenario-tree models, statistical models or machine-learning methods. The resulting risk estimates can then be used to stratify farms, rank them for inspection or assign different selection probabilities, for example through probability-proportional-to-risk sampling (12, 13). Scenario-tree models provide a structured framework for combining predefined risk pathways, probabilities and expert knowledge in veterinary surveillance (14). In the present study, we aimed to develop an explicitly data-driven risk-ranking method based on routinely recorded administrative and veterinary variables, without introducing additional expert-assigned weights, scores or risk categories. Extreme Gradient Boosting (XGBoost) was considered suitable for this purpose because the available data comprised heterogeneous predictors with potentially nonlinear associations and interactions, as well as missing values. As a tree-boosting method, XGBoost can model such relationships and generate a continuous score for ranking farms without requiring all thresholds and interaction structures to be specified in advance (15). The resulting model was intended to provide a risk-estimation and ranking component that complements, rather than replaces, the existing risk-based and expert-informed control system.

Against this background, the primary objective of this study was to develop a deliberately data-driven model based on routinely recorded administrative and veterinary variables to rank livestock farms according to their predicted risk of a recorded positive animal-welfare inspection outcome. Our main question was: Which routinely recorded farm and animal-related characteristics are associated with a higher predicted risk of a recorded positive animal-welfare inspection outcome and could therefore support the prioritization of farms for official veterinary inspection? The model is intended to complement and support the development of the inspection plan by providing an additional, data-driven basis for prioritizing farms while maintaining the required broader sampling of farms. Farms with higher predicted risks could therefore be given higher priority within the overall selection process, supporting a more targeted allocation of available inspection resources. The model is intended as a decision-support tool and would not automatically trigger additional monitoring or inspections.

## 2 Materials and methods

The analytical dataset comprised 3,122 aggregated farm-year inspection records from official animal welfare inspections conducted in Styria between 2019 and 2023. These records were used to develop and evaluate an Extreme Gradient Boosting (XGBoost) model for the risk-based prioritization of livestock farms for official inspection.

Official animal welfare inspections in Styria are conducted within a risk-based and probabilistic control system. Farm selection is not based exclusively on animal-welfare-related criteria: for administrative efficiency, animal welfare inspections may be combined with other official controls, including feed and milk hygiene controls. Selection also incorporates available administrative information and professional assessment by the competent authorities. The sampling frame comprised all eligible livestock farms, and no farm category was systematically excluded from selection; however, selection probabilities were unequal. Because individual farm-level inclusion probabilities could not be reconstructed no inverse-probability weighting or other formal correction for selection bias was applied.

Inspections were carried out by official veterinarians using the animal welfare manuals and checklists issued by the Federal Ministry of Labor, Social Affairs, Health, Care, and Consumer Protection (16). To promote a consistent and objective assessment across inspections, official veterinarians in Styria routinely participate in calibration training sessions for animal welfare inspections. These sessions aimed to harmonize the application and interpretation of the relevant assessment criteria and thereby reduce variation between inspectors. The resulting inspection outcomes were used as the basis for defining the binary target variable in the present study. A farm-year observation was classified as positive when an animal welfare deficiency was documented during an official inspection or when a criminal complaint was filed as a consequence of the inspection. The number of farms with animal welfare violations, categorized according to the respective assessment categories, is presented in Supplementary Table S1. Farms without an observed inspection outcome were not included as negative cases.

A gradient boosting machine learning model was used to rank farms according to their predicted probability of a positive recorded inspection outcome. The model was intended to support prioritization of farms for official inspection and not to estimate the population prevalence of underlying animal welfare violations. The approach is based on an ensemble of decision trees that are fitted sequentially, where each tree optimizes the residuals of the preceding ensemble, thereby improving predictive performance and reducing variance compared with single-tree models. Model hyperparameters were tuned using a cross-validated grid search, shown in Supplementary Figure S2. Hyperparameter tuning was performed exclusively on the training data using cross-validation. The test set was not used at any stage of tuning procedure. All analyses were performed in R version 4.3.3, and model fitting was conducted using the xgboost package (version 1.7.11.1) (17, 18).

Model performance was assessed using the area under the receiver operating characteristic curve (AUC-ROC), the Matthews Correlation Coefficient (MCC) and a gain plot (19, 20). The ROC represents the true positive rate (sensitivity) versus the false positive rate (1-specificity). The value ranges from 0 to 1. An AUC of 0.5 suggests no discrimination, meaning that the model would perform as well as a random selection. A value close to 1 would represent a theoretically perfect model, whereas a value close to 0 indicates that the model systematically predicts the wrong class. The Matthews Correlation Coefficient (MCC), on the other hand is a metric used to evaluate the quality of binary classifications. This value ranges from −1 to 1. As with the AUC, a score of 1 would represent a theoretically perfect model. Values close to 0 correspond to random performance, while −1 indicates total disagreement between predictions and observations. Provided is also average precision (AP) and confidence intervals for each metric. Average precision was calculated by ranking observations according to their predicted probability of the positive class and computing precision at each positive observation. AP corresponds to the mean precision across these ranks. Confidence intervals (95% CI) were estimated using 5,000 non-parametric percentile-bootstrap samples. Farms were used as the resampling unit where repeated observations per farm were present; otherwise, individual observations were resampled.

The area under the ROC curve (AUC-ROC) is insensitive to class imbalance, whereas the Matthews Correlation Coefficient (MCC) is prevalence-dependent and is not directly comparable across datasets with substantially different class distributions. However, unlike accuracy or F1-score, it does only assign a high score when a classifier performs well on both classes and provides a balanced summary of all four cells of the confusion matrix. Both metrics were therefore considered appropriate for the present study, where non-compliant farms (8.1%) were strongly underrepresented relative to compliant farms (91.9%). Because the intended application was to prioritize farms for inspection, random selection was used as an operational benchmark for the ranking task. The random-selection reference represents the expected cumulative identification of positive inspection outcomes when farms are selected without risk-based prioritization. A gain plot (cumulative gains chart) shows the cumulative proportion of positive inspection outcomes identified by a model as a function of the proportion of the population, ranked by predicted probability. It compares model-based prioritization with random selection and illustrates the proportion of recorded positive inspection outcomes identified among the top-ranked farms.

To this end, characteristics of each farm and species that could support the ranking of farms according to their risk of a positive recorded inspection outcome were included as model features. All reported features were included, and feature selection was not performed as a pre-processing step; instead, the internal feature selection mechanism of XGBoost was relied upon to identify the most relevant predictors during model training. The characteristics were based on data from the Consumer Health Information System (*Verbrauchergesundheitsinformationssystem* (VIS)) and the fallen stock rendering plant (*Tierkörperverwertung* (TKV)). The available data were split into 85% for training the model and 15% for testing. This ratio was chosen to ensure a sufficient number of training observations given the relatively low prevalence of non-compliant outcomes, and because farms inspected across multiple years were assigned exclusively to either the training or the test set to prevent data leakage. This farm-level partitioning reduced the number of independent units available for testing and motivated the use of a larger training proportion. Therefore, a total of 2,647 aggregated inspection results from 2,387 unique farms were used for training and 475 inspection results from 421 unique farms for testing. A table showing train and test split as well as the number of farms with specific set of inspections results is represented in the Supplementary Table S3.

To facilitate *post hoc* model interpretation, SHapley Additive exPlanations (SHAP) values were calculated. Global feature importance was summarized using the mean absolute SHAP values, while the sign of the SHAP values indicated whether a given feature value contributed to an increase or decrease in the model-predicted probability for an individual observation.

### 2.1 Features

The model included six main categories of influencing factors: farm information, production type, dispensing of antibiotics, data from the ante-mortem and post-mortem slaughterhouse inspection, fallen/dead stock rendering plant and density of the location of veterinary practices. The data were obtained from administrative databases maintained by the respective data custodians. The databases are subject to regular quality assurance and validation procedures conducted by the responsible data providers. Before model development, descriptive data checks were performed to identify implausible values, inconsistent entries, unusual distributions, and potential reporting errors. No duplicated records or systematic errors were identified, the available data were used, without introducing *ad hoc* corrections to individual records.

Features with only two possible values (such as ‘yes’ or ‘no’) were encoded as 0 for ‘no’ (false) and 1 for ‘yes’ (true). Features with non-numeric values were ‘one-hot-encoded’ into multiple features, one for each possible value. After preprocessing and one-hot encoding, the final dataset contained 80 model features. Descriptive statistics for all features are provided in Supplementary Table S4. If no or insufficient information was available for a feature, its value was coded as NA (“not available”). The rules used to determine when values were coded as NA are described in the Supplementary Table S5. No farms were excluded because of missing feature values; instead, missing values were retained as NA, the modeling approach handled these missing values directly, allowing missingness to potentially contribute to model predictions. In summary, the model examined various features across all six subcategories which are listed in Table 1. The target variable, inspection result, was paired with feature values from the previous year for each inspection, unless otherwise specified. Although some features are species-specific, all features were included in a single general model. Farms are selected for inspection based on their overall risk across all species and are subsequently inspected for every species present on the farm. A general model does increase practicability compared to species-specific models. Nevertheless, sensitivity analyses of model performance using species-specific models were also conducted and are reported in the Supplementary Table S6. Detailed descriptions, summary statistics, and missingness information for all model features are provided in Supplementary Tables S4 and S5. The features are briefly described below.

**Table 1:** Overview of features included in the model as potential indicators of animal welfare violations.

Feature group	Species/Category	Feature	Data type
Production type	Cattle	No cattle production	Binary
		Calf fattening	Binary
		Cattle fattening/beef	Binary
		Dairy	Binary
		Suckler cow husbandry/fattening	Binary
		Alpine cattle pasture	Binary
		Livestock units cattle	Continuous
	Pigs	No pig production	Binary
		Pig rearing gilts	Binary
		Pig fattening	Binary
		Pig breeding	Binary
		No pig stock during evaluation period	Binary
		Combined farrow-to-finish	Binary
		Free range pig farming	Binary
		Livestock units pigs	Continuous
	Poultry	No poultry production	Binary
		Laying hens	Binary
		Pullets (rearing)	Binary
		Poultry fattening/broilers	Binary
		Chicken	Binary
		Turkey	Binary
		Undefined poultry	Binary
		Free range poultry farming	Binary
		Organic poultry farming	Binary
		Livestock units poultry	Continuous
	Sheep	No sheep production	Binary
		Livestock units sheep	Continuous
	Goat	No goat production	Binary
		Livestock units goat	Continuous
	Horse	No horse production	Binary
		Livestock units horse	Continuous
Farm information		Single Application Form (CAP subsidies)	Binary
		Member of Animal Health Service	Binary
		Maximum age of farmer	Count
		Number of managers	Count
		Maximum number of farms of each associated person	Count
		Maximum distance between all farms of each associated person	Continuous
		Distance farm to farmer’s home address	Continuous
		Maximum distance from farm to all people home address	Continuous
Ante-mortem and post-mortem Slaughterhouse inspection	Cattle, pigs, poultry, sheep, goat, horse	Relative number of C (animal welfare violation)	Ratio
		Relative number of D061* (polyarthritis)	Ratio
		Relative number of D164* (multiple abscess)	Ratio
		Relative number of D164a* (other findings)	Ratio
		Relative number of E167# (pneumonia)	Ratio
		Relative number of E168# (pericarditis)	Ratio
		Relative number of E169# (pleuritis)	Ratio
		Relative number of E169a# (peritonitis)	Ratio
		Relative number of E169gs# (adhesion/sticking of entrails)	Ratio
Fallen/dead stock rendering plant	Cattle	Relative number of abortions/stillbirths (cattle)	Ratio
		Relative number of dead calves (<6 months)	Ratio
		Relative number of dead youngstock cattle (6–24 months)	Ratio
		Relative number of dead adult cattle (>24 months)	Ratio
		Relative number of dead cattle unknown age	Ratio
	Pigs	Relative number of dead pigs	Ratio
		Relative number of dead piglets	Ratio
	Poultry	Ratio dead animals to stock figure (in LSU)	Ratio
	Sheep	Relative number of dead sheep	Ratio
	Goat	Relative number of dead goats	Ratio
	Horse	Relative number of dead horses	Ratio
Veterinary practices		Number of animal health service veterinarians per district	Continuous ratio
		Number of animal health service veterinarians per municipality	Continuous ratio
Antibiotics	Cattle	Total dispensed antibiotic cattle (nDDDvet)	Continuous ratio
		Dispensed antibiotics Category B cattle (nDDDvet)	Continuous ratio
		Dispensed antibiotics Category C cattle (nDDDvet)	Continuous ratio
		Dispensed antibiotics Category D cattle (nDDDvet)	Continuous ratio
	Pigs	Total dispensed antibiotic pigs (nDDDvet)	Continuous ratio
		Dispensed antibiotics Category B pigs (nDDDvet)	Continuous ratio
		Dispensed antibiotics Category C pigs (nDDDvet)	Continuous ratio
		Dispensed antibiotics Category D pigs (nDDDvet)	Continuous ratio

*SFU category D = carcass condemnation. #SFU category E = pathological findings, but limited to certain organs, carcass not condemned. DDDvet, defined daily dose for veterinary medicine; CAP, common agricultural policy.

#### 2.1.1 Farm information

Various details about the farmers themselves are available for each farm. One potential factor influencing the likelihood of an animal welfare violation was considered to be membership of the Animal Health Service (*Tiergesundheitsdienst* (TGD)). The Animal Health Service aims to prevent animal diseases and improve the overall health and welfare on the farm. TGD veterinarians are only permitted to dispense certain antibiotics to farmers who are also members of the TGD and who have completed the appropriate medicine administration and handling courses (21). Important to note is that according to the List of veterinary medicinal products approved pursuant to Section 2(2) of the Veterinary Medicinal Products Application Regulation 2010 (Federal Law Gazette II No. 259/2010), certain antibiotic-containing veterinary medicinal products may also be dispensed to animal holdings that are not members of the TGD, provided that the applicable legal requirements are met (22).

Another possible influencing factor could be the Single Application Form (SAF, *Mehrfachantrag*), an annual application Austrian farmers can submit to claim EU agricultural subsidies under the Common Agricultural Policy (CAP) including area-based aid and subsidies for improved animal welfare practices linked to compliance with certain management and husbandry standards (23). To do so, farmers must provide information on land area, land use, livestock numbers, and participation in agri-environmental and animal welfare programs. The application is typically submitted electronically within legally prescribed deadlines. Failure to submit SAF excludes access to these CAP-based financial supports.

Since farm-specific characteristics, such as the physical location and the number of individuals involved in farm operations, may be associated with the likelihood of animal welfare violations, the number of managers per farm, the maximum number of farms to all farmers and the distance from the farm to the home address were further features of interest. If a farm had more than one manager, the maximum distance to a residential address was considered. If a farmer owned more than one farm, the maximum distance between all farms was taken into account. The maximum age of farmers was the last characteristic included under this category. For this set of features there is no historical data available, which means the state at modeling is used for values of features for all years.

#### 2.1.2 Production type

The number of animals was calculated for each species, measured in livestock units (LSU, *Großvieheinheit* (GVE)), the standard EU conversion unit used to compare different farm animals based on their liveweight (24). For cattle, information on the type of farm (dairy, calf fattening, cattle fattening/beef, suckler cow husbandry/fattening) and alpine pasturing registration was obtained, partly based on animal movement data. In pig production, a distinction was made between rearing gilts, breeding, fattening, and combined farrow-to-finish systems. For poultry, three production types are available: laying hens, pullets (rearing) and broilers. These were also classified according to whether they reared chickens or turkeys, or whether the species was not specified. For pigs and poultry, an additional factor considered was whether the farm engaged in free-range farming. For horses, goats, and sheep, no further distinction was made between the production systems. Only the presence of these species and the herd size in LSU were considered.

#### 2.1.3 Antibiotics

Quantities of veterinary antibiotics dispensed by the herd veterinarians to the farmer are documented in the PHAROS database (25). The dose-based unit of measurement DDDvet (Defined Daily Dose for animals) as defined by EMA (European Medicines Agency) is used (26). The total defined daily doses of antibiotics dispensed (adjusted for herd size) per year are defined for each farm, enabling comparisons of these characteristics between farms regardless of their size (27, 28). EMA categorizes antibiotics into four groups: Category A antibiotics are not licensed for use in veterinary medicine and are never permitted for use in food-producing animals and were therefore not included in this model. Categories B and C are subject to restrictions, such as compulsory sensitivity testing (29). While category D includes antibiotics used as first line treatments, such as penicillins, tetracyclines and sulfonamide/trimethoprim combinations (30). Information about antibiotics becomes available with higher delay compared to data from other features. Therefore, each inspection result is matched with antibiotics data from 2 years prior to the inspection.

#### 2.1.4 Ante-mortem and post-mortem slaughterhouse inspection

The data on slaughter and meat inspection (*Schlachttier- und Fleischuntersuchung* (SFU)) provide information on the number of inspections carried out and the number of identified findings. Inspectors must categorize their findings using a standardized SFU-code (A, B, C, D, E) (31, 32). Categories A and B, which are not included in this model, refer to animal diseases that are listed as OIE/WOAH diseases, such as foot-and-mouth disease or tuberculosis. These classifications were not taken into account because they involve highly infectious notifiable and not notifiable diseases that are not considered to be directly related to animal welfare violations. Category C describes obvious animal welfare violations, which are primarily visible at ante-mortem inspection. Post-mortem, Category D is used when the entire carcass is condemned and category E when only certain organs are unfit for human consumption. Each category is assigned a number to specify the findings. For the model, all category C entries were combined due to the low number recorded. All category C, D & E entries were potentially expected to indicate an animal welfare violation and were therefore taken into account in the modeling and are listed in Table 1.

#### 2.1.5 Fallen/dead stock rendering plant

Rendering plants also maintain important data. The number of fallen/dead stock in each category is collated for each farm. For horses, goats and sheep, the number of dead animals is divided by the herd size. For poultry, weight in livestock units is a more accurate measurement than counting individuals. For these species, a ratio of dead animals to livestock numbers is calculated using LSU. For pigs, the number is divided into adult pigs and piglets. Due to the more detailed database for cattle, four subgroups are used: abortion/stillbirth; calves (<6 months old); youngstock (6–24 months); and adult cattle (>24 months). Unknown age of the animal is indicated accordingly.

#### 2.1.6 Veterinary practices

Information about veterinary care was included in the model. The regional density of TGD veterinarians in a district or municipality was taken into account. By definition, to be veterinary members of the TGD, these practitioners are routinely (although perhaps not exclusively) treating farm animals. Therefore, the number of TGD veterinarians, whose practice address was located in the same region as the farm, was divided by the total number of farms in that region.

### 2.2 Feature importance—SHAP

The SHAP (Shapley Additive Explanations) method is used to interpret the results of machine learning models by assigning a contribution value to each feature. SHAP values quantify the contribution of each feature to the model prediction. A positive SHAP value indicates that the corresponding feature value increases the prediction. In this case, a higher SHAP value indicates a higher predicted probability of animal welfare violations. A negative SHAP value indicates that the corresponding feature value decreases the prediction. The larger the absolute negative value, the greater the negative contribution of the feature to the prediction; similarly, the larger the positive value, the greater the positive contribution. Feature importance is calculated as the mean of absolute SHAP values (MSV). SHAP was chosen because it provides a consistent additive decomposition of individual model predictions and can summarize both the magnitude and direction of feature contributions for a tree-based model. In this study, SHAP was used for *post-hoc* interpretation and communication of model behavior, not as a causal analysis or as a stand-alone rule for selecting farms. When predictors are correlated or structurally linked, SHAP contributions may be shared between variables; therefore, the reported rankings should be interpreted as model-based attributions rather than independent effects.

## 3 Results

### 3.1 Model performance

Results showed an AUC (Area under the curve) of 0.75 (95%-CI [0.66, 0.82]), MCC (Matthews Correlation Coefficient) of 0.25 (95%-CI [0.19, 0.37]) and AP (Average Precision) of 0.19 (95%-CI [0.11–0.31]) for the test set. The corresponding precision-recall curve is shown in Supplementary Figure S1. As shown in Figure 1, a selection of livestock farms with the trained model outperforms random selection also in terms of gain. The gray curve demonstrates a theoretical optimal model. Random selection would yield a corresponding proportion of animal welfare violations, resulting in points lying along the diagonal. The curve of the model is located above the diagonal within the shaded area, indicating that the selection strategy identifies a higher proportion of positive cases than expected under random sampling.

**Figure 1:**
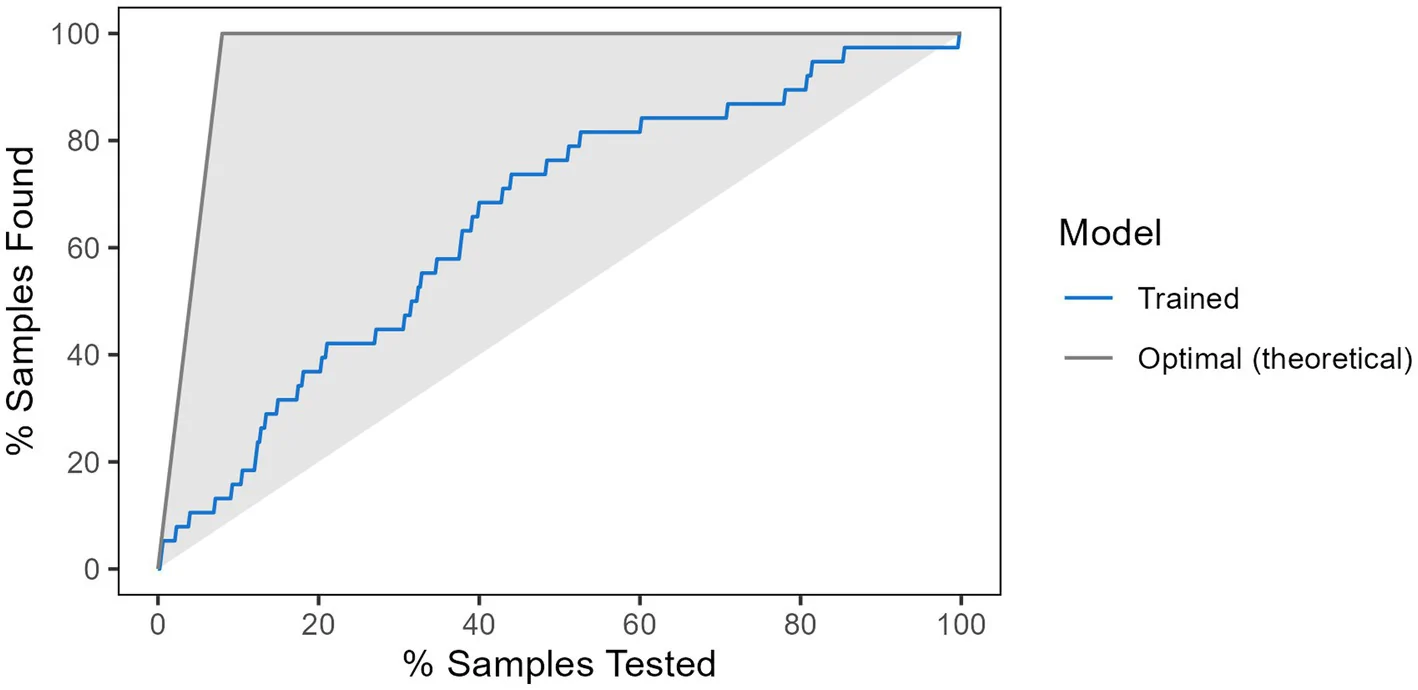
Gain plot showing the proportion of farm violations detected by the trained model (blue) relative to the proportion of tested samples, compared with a theoretical optimal model (gray).

### 3.2 Feature importance and interpretation with SHAP values

Figure 2 lists the top 15 features that most strongly influenced the model’s prediction of animal welfare violations according to MSV. Features with the highest MSV are listed at the top. Additional SHAP plots for lower-ranked features are provided in Supplementary Figures S2 and S3. Due to their low relevance for the prediction, the remaining features are not shown in the SHAP diagrams. The feature value is represented by different shades of purple/yellow: dark purple indicates a high feature value, while light yellow points represent lower feature values. If this information is not available, the dots are displayed in gray. Each dot represents an observation. To enhance readability, the values were plotted with vertical offsets.

**Figure 2:**
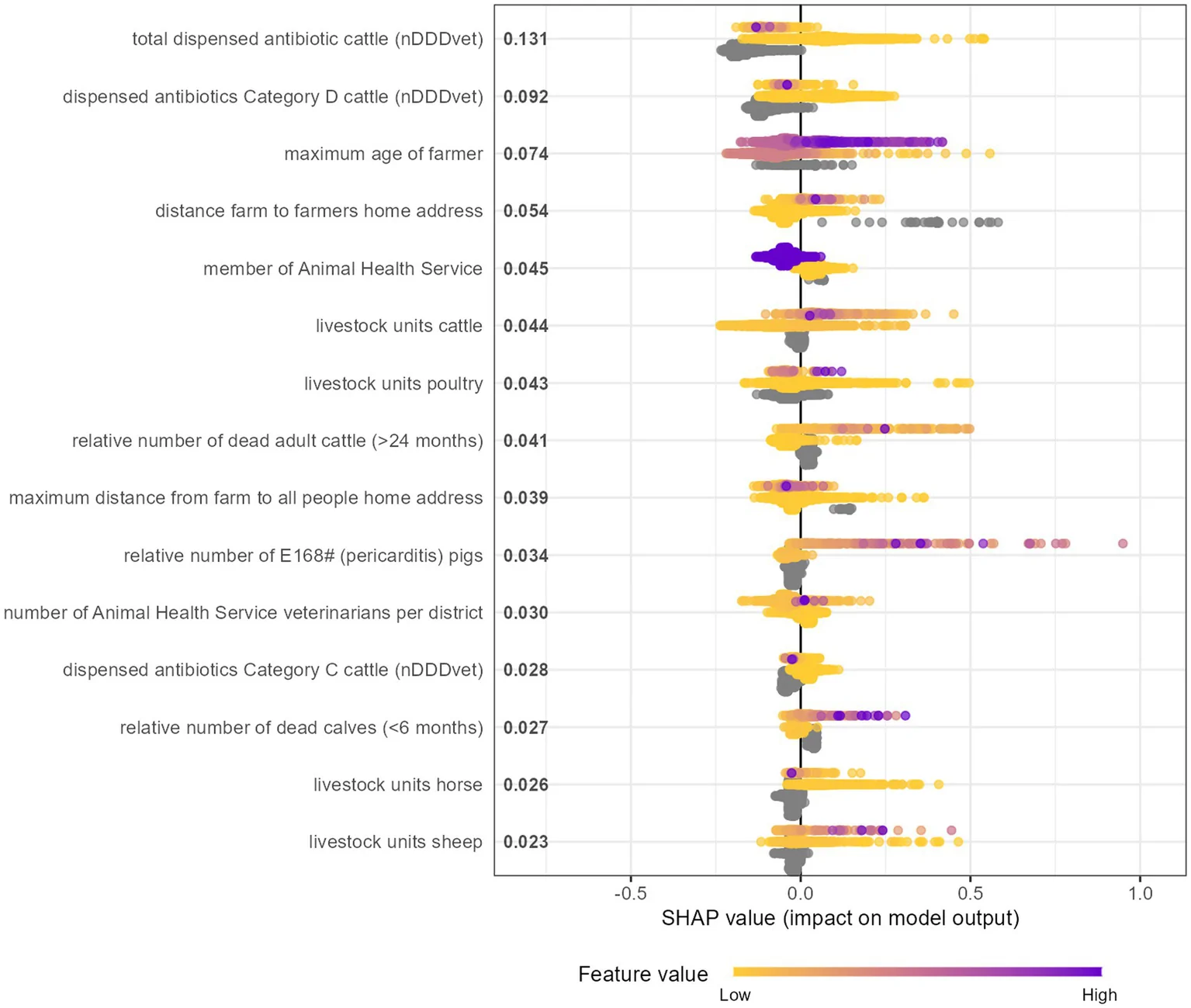
SHAP summary plot showing the contribution of the 15 most influential features to model predictions. Features are ordered by decreasing mean absolute SHAP value. Each point represents a farm. SHAP, Shapley Additive Explanations. DDDvet = Defined Daily Dose for veterinary medicine# SFU category E = pathological findings, but limited to certain organs, carcass not condemned.

#### 3.2.1 Most relevant features

Five out of the six main feature groups are listed in the top ten, i.e., the most relevant features for predicting animal welfare violations, with only information about veterinarian density falling outside this range to number eleven (Figure 2). The features with the highest mean absolute SHAP values (MSV) were the total amount of dispensed antibiotics for use in cattle production (MSV 0.131), the amount of dispensed antibiotics in category D for cattle (MSV 0.092), maximum age of the farmer (MSV 0.074), maximum distance from the farm to the farmer’s residential address (MSV 0.054), and Animal Health Service membership (MSV 0.045). These are followed by LSU of cattle (MSV 0.044) and LSU of poultry farms (MSV 0.043) as well as the number of dead adult cattle removed from the farm by the rendering plant (MSV 0.041). The maximum distance between the residential address and the farm, when a farm has more than one manager (MSV 0.039) and the presence of pericarditis in pigs at the slaughterhouse (MSV 0.034) are also among the most relevant features. Animal Health Service membership is a feature with a particularly clear result: non-membership was associated with positive SHAP values, corresponding to a higher model-predicted probability of an animal welfare violation, whereas membership was associated with negative SHAP values and a lower predicted probability (Figure 2). The model-predicted probability of animal welfare violations is higher in association with the presence of pericarditis in pigs, a greater number of cattle on the farm (LSU cattle), and a greater number of fallen adult cattle sent for rendering. While a greater distance between the farm and the residential address is associated with a higher model-predicted probability of a violation, missing (i.e., unknown) values for this characteristic also associated with a higher model-predicted probability. In contrast, a higher amount of dispensed antibiotics (total and category D) on cattle farms is associated with a lower model-predicted probability of an animal welfare violation. It is important to note, however, that there were relatively few cattle farms with high antibiotic use. Other features, such as the farmer age, LSU poultry, and the maximum distance of all residential addresses from a farm when more than one farmer is involved, show a more diffuse distribution.

#### 3.2.2 Density of veterinarian practices

The characteristics describing the association between the density of veterinary practices and the model-predicted probability of animal welfare violations is not among the top ten but is listed as eleventh (MSV 0.030) (Figure 2). A high density of livestock veterinarians in a district is associated with both positive and negative SHAP values, indicating that its relationship with the model-predicted probability varied across observations. Similarly, a low density of livestock veterinarians was associated with both higher and lower model-predicted probabilities.

#### 3.2.3 Farm information and production type

In addition to Animal Health Service membership, application of the SAF (MSV 0.015) and access for cattle to alpine pastures (MSV 0.007) are also associated with a lower model-predicted probability of an animal welfare violation, while the lack of application for these subsidies or lack of access to alpine pastures is associated with a higher model-predicted probability (Supplementary Figure S2).

#### 3.2.4 Antibiotics

High levels of total antibiotics dispensed (MSV 0.131) to cattle farmers, particularly Category D (0.092) and, to a lesser extent, Category C (MSV 0.028), are associated with a lower model-predicted probability of animal welfare violations (Figure 2). This association was also observed for the same features if no data was available. However, as shown in Supplementary Figures S2 and S3, higher dispensing of category B antibiotics in cattle (MSV 0.011), and higher levels of dispensing of all antibiotics in pigs is associated with a higher model-predicted probability of an animal welfare violation: categories B (MSV 0.009), C (MSV 0.005) and D antibiotics in pigs (MSV 0.005).

#### 3.2.5 Quantity of livestock units and dead stock

In addition to the LSU of cattle and poultry, other animal species also show uneven distribution. Although not entirely clear, a higher LSU of cattle, poultry and sheep tends to be associated with a higher model-predicted probability of animal welfare violations. As shown in Figure 2 and Supplementary Figures S2 and S3, a higher number of dead animals sent for rendering is generally associated with a higher model-predicted probability of an animal welfare violation, except for the number of dead goats (MSV 0.006) (Supplementary Figure S3). For cattle, a distinction could be made for the different age categories: specifically, a high mortality rate among calves leads to a higher model-predicted probability of animal welfare violations on a farm (MSV 0.027) (Figure 2).

#### 3.2.6 Findings of ante-mortem and post-mortem slaughterhouse inspection

As seen in Figure 2, Supplementary Figures S2 and S3, findings in post-mortem meat inspections of cattle and pigs (in particular, pericarditis, MSV 0.034) are associated with a higher model-predicted probability of an animal welfare violation. However, seen in Supplementary Figure S2, the presence of peritonitis (169a) in pigs decreases the model-predicted probability (MSV 0.011).

## 4 Discussion

This study highlights the potential of routinely collected data for the prediction of animal welfare violations. The results suggest that animal welfare outcomes are influenced by a range of interconnected factors and underscore the value of combining information from different data sources. Although SHAP facilitates the identification of features that are relevant to the model’s predictions, the dependencies and interactions among these features cannot be explicitly represented within this SHAP analysis itself. These associations should not be interpreted as evidence of causal relationships. The following sections discuss the most influential predictors identified by the model and their implications in relation to existing literature.

### 4.1 Farm information

There is little research on how general farmer data influences the likelihood of animal welfare violations. However, farmers’ physical and mental health is known to often play an important role in influencing animal welfare (33). Although these factors can have a major impact, data protection regulations or lack of data accessibility make it nearly impossible to include them in statistics or studies (9). Nevertheless, the results showed that even the limited data available had a significant influence on the characteristic target values. With four farm-related characteristics among the top ten most influential factors, these data have a greater impact predicting animal welfare violations than originally assumed. Animal Health Service membership was clearly demonstrated to show a lower model-predicted probability of an animal welfare violation (negative SHAP value) when a farmer was an Animal Health Service member (feature value = purple). Conversely, animal welfare violation is associated with a higher model-predicted probability (positive SHAP value) when the farmer is not a member (feature value = yellow). The feature SAF showed similar results, possibly indicating that farmers who actively apply for these EU subsidies also have better overall management and therefore a lower predicted probability of animal welfare violations.

The age of the farmer is also an important factor in this model. Older age tended to increase the predicted probability of animal welfare violations. However, the results are not as clear-cut as for Animal Health Service membership. In this model, young farmers were associated with a higher risk of committing animal welfare violations and middle age was more neutral. This would also be consistent with the literature (34–36). On the one hand, older farmers have more experience than younger ones. A review of farmers’ views on animal welfare describes how older farmers have more knowledge about animal diseases and a higher empathy score, making them more sensitive to animal pain and welfare (34). On the other hand, animal welfare is often related to the farmer’s own physical and mental well-being. High occupational well-being, low stress, low loneliness, farmer optimism and determination to continue production, are associated with a high animal welfare index (33). Although age had a major impact on the model, it may not always reflect the real situation on the farm: sometimes an older farmer is officially retired and no longer registered as the owner but still manages the farm on a daily basis.

The model-predicted probability of a welfare violation increased with the distance between the place of residence and the farm, and also when no information on residential address was available. When more than one farmer was listed as an associated person, the results were less clear. A greater maximum distance from the place of residence of all farm-associated people had both positive and negative SHAP values, while a shorter maximum distance to the farm appeared to reduce the predicted probability of violations. If a farmer lives close to or on their farm, it is easier for them to monitor animal health and welfare. On the other hand, distance may also prevent operational blindness, also called “farm blindness” (37). While farmers who live on their farms may fall into a routine, farmers who only work there and live elsewhere can maintain some objective distance and therefore may have a fresher perspective. Missing values occurred when a farm was still registered in the VIS, but was no longer actively engaged in animal husbandry, sometimes due to the fact that a farmer has been prohibited from keeping animals. Another reason could be that the farmer never filled in this information for the farm, which also could be explained by a lack of general farm management and organizational skills and would therefore be an explanation for an increased risk for animal welfare violation.

### 4.2 Production type

The herd size of a farm (i.e., LSU) especially for cattle and, to a lesser extent, for poultry is very important for the model used to predict animal welfare. Compared to many other countries, Austria has small-scaled livestock farms, with an average of only 36 head of cattle or 119 pigs (3, 38). It is often assumed that smaller farms have fewer animals to care for and therefore, in theory, farmers can spend more time with each individual animal while larger farms are often viewed as industrial, intensive production facilities where animals are just numbers and animal welfare is not a priority (39–41). However, previous studies have shown that animal welfare does not depend solely on farm size (38, 40, 64). Larger farms have more animals to care for, but they often have better management systems to monitor and measure animal health and welfare and often more staff on site, while smaller farms may focus more on the individual animal but often have fewer resources to change operating systems to improve the general animal welfare. Therefore, animal welfare is influenced more by the options available, the opportunities for improvement and farmers’ attitude toward animal welfare and husbandry than by herd size per se (42, 43).

### 4.3 Antibiotics

The use of antibiotics in farm animals is a topic of great interest, particularly regarding antibiotic resistance (44). However, treating sick animals is also a key aspect of animal welfare. Quantifying AMU (antimicrobial usage) in livestock farming is complex and therefore difficult to compare (45, 46). It is important to note that, at present, the antibiotic amounts recorded in the Austrian statistics and in this model only represent those prescribed and dispensed to farmers by veterinarians. Antibiotics directly administered by veterinarians (as is common in cattle production) are not included in these datasets and dispensed antibiotics may not have been used by the farmers for the prescribed purpose.

In this model, the prescription and dispensing of antibiotics for use in cattle and pigs leads to different predictive outcomes, which may be due to the extremely different types of veterinary treatment (individual versus group treatments, administration by the herd veterinarians (which is, therefore, not reported to the veterinary authorities at the time of this data collection) versus dispensing for administration by the farmer) common in these animal production systems. While high levels of total antibiotics dispensed for use in cattle (both total and Category D) reduced the predicted probability of an animal welfare violation, the opposite was true for pigs. Cattle and pigs differ significantly in their husbandry practices. Pigs are often kept in larger groups and treated accordingly, while cattle, particularly dairy cows, are more frequently treated individually. This could indicate that high antibiotic prescription rates in pigs are either detrimental to animal health or (more likely) indicative of a substantial, possibly long-term, herd health problem and thus increase risk for animal welfare violations. In contrast, antibiotic use in cattle is predominantly individualized, meaning that higher treatment levels may be associated with targeted, indication-based therapy and may therefore have a positive effect on animal health. On the other hand, improved animal welfare is often associated with reduced antibiotic usage (47). This also aligns with the results of this model, as a lack of data on antibiotic dispensing in cattle reduces the predicted likelihood of animal welfare violations. This could indicate that good health management on cattle farms could prevent the necessity for antibiotic use, or it could indicate that a large proportion of antibiotic treatments on cattle farms are administered by the herd veterinarian themselves (therefore, not requiring reporting to the PHAROS database under the current legislation), meaning that the veterinarian is frequently on farm. The predictive value of TGD membership for antibiotic dispensing may be partly related to the fact that the dispensing and administration of certain injectable veterinary medicinal products within the TGD are subject to completion of a specific application course. TGD members who have completed the required course may therefore have access to medicinal products for injectable administration. At the same time, the official list of approved veterinary medicinal products includes several antimicrobial products that may also be dispensed to non-TGD farms under specific circumstances (22). A cross-tabulation of TGD membership and the corresponding antibiotic categories is provided in the Supplementary Table S7. To summarize, antibiotic use or dispensing records alone cannot be used to predict the probability of welfare violations or issues with animal health.

### 4.4 Ante-mortem and post-mortem slaughterhouse inspection data (SFU)

Ante- and post-mortem slaughterhouse inspections are crucial for food safety. Abnormal findings, however, may also indicate animal welfare violations. There is growing interest in using this data to predict animal welfare violations (48–51). In this study, pericarditis in pigs proved to be highly predictive, while other findings appeared to have less influence than expected. In practice, SFU findings are collected by official veterinarians and meat inspection assistants. Although criteria are established for each abnormality, the results from the same carcass can differ between inspectors and different slaughterhouses can also record extremely heterogeneous results (52–56). Due to this subjectivity, SFU findings can differ substantially from the true condition of the carcass. Another factor is the number of available findings: category C findings, although particularly relevant to animal welfare violations, were too few to analyze separately. As post-mortem dissections and meat inspection primarily focus on identifying abnormalities affecting food safety, animal welfare findings are considered less relevant and, even if present, are rarely documented. The low number of findings could also explain why higher peritonitis findings in pigs was associated with a reduced model-predicted probability of animal welfare violations. A previous Austrian study identified peritonitis as the rarest finding, with a prevalence of only 0.1% (57). It is therefore possible that, within the present data set, farms without recorded animal welfare violations experienced occasional periods with elevated peritonitis incidence, resulting in a misleading inverse association in the model. Furthermore, the MSV of this feature was relatively low at 0.011. By contrast, a Danish study found that pericarditis detected at meat inspection was weakly correlated with noticeable health findings on farm, so it is possible that while welfare issues were present in farms with this issue, the farmer was not necessarily fully aware of them (58). However, the authors of this Danish study also stated that they believed pericarditis was often underdiagnosed at meat inspection.

### 4.5 Fallen/dead stock rendering plant

In cattle, the number of deadstock animals over 24 months of age sent to the rendering plant was associated with an increased likelihood of animal welfare violations. High mortality can indicate poor animal health or general farm management problems (59, 60). Calf and pig mortality were also among the 20 most important factors for predicting animal welfare violations in this model. Consistent with previous studies, cow and calf mortality are considered important animal welfare indicators (61, 62). In addition to the mortality rate, necropsy findings can provide valuable information on animal welfare. Post-mortem dissections are frequently performed to determine the cause of death and to rule out relevant animal diseases. In some cases, dissections are also carried out in response to animal welfare concerns. The results are forwarded to the district authority responsible for further investigation or the initiation of criminal proceedings While informal training of employees to recognize animal welfare issues exists at a number of rendering plants in Austria, there is currently no standardized system in place for recording animal welfare parameters (63). Standardized procedures and protocols for rendering plant employees could provide additional useful information.

### 4.6 Veterinary practices

Although the density of veterinary practices is not among the top ten factors, it ranks eleventh for relevance in the model. A higher density of veterinary practices treating livestock would be expected to decrease the predicted likelihood of animal welfare violations by improving access to veterinary care. However, such an association was not observed in the present analysis. The veterinarians included in this model are all members of the Animal Health Service, treat food-producing animals, and have contracts with their farmers that include, among other things, an annual review of animal health and welfare. Most farmers who are Animal Health Service members have attended the relevant medication training courses, are authorized to perform follow-up treatments themselves, and can therefore administer and inject intramuscular and subcutaneous medications as prescribed by a veterinarian. Due to the close cooperation between farmers and veterinarians, as well as the ability for farmers to perform follow-up treatments themselves, membership of the Animal Health Service seems more important than the actual number of veterinarians in a district.

### 4.7 Selection bias

The outcome was observable only when farms came to the attention of the authorities through the existing Styrian control and surveillance system. It therefore represents a recorded animal welfare deficiency or associated criminal complaint, rather than all underlying violations in the farm population. Because farm selection is probabilistic but risk-based and incorporates multiple control purposes, administrative information and professional assessment, the inspected sample may differ systematically from the wider farm population.

Although all eligible livestock farms are included in the sampling frame, selection probabilities were unequal and the inspected sample cannot be assumed to be representative of all Styrian livestock farms. Farm-specific inclusion probabilities could not be reconstructed, and no formal correction for selection bias was applied. Farms without an observed outcome were not treated as negative cases; consequently, violations on uninspected farms may remain undetected. Passive surveillance, such as public complaints, may partially mitigate but cannot resolve this limitation.

The model should therefore be interpreted as a risk-ranking tool for prioritizing inspections within the existing control and surveillance system, rather than as an estimate of the unconditional probability or population prevalence of animal welfare violations.

### 4.8 Limitations

This study has several limitations that should be considered when interpreting the results. The model is based on routinely collected administrative data, which may include inconsistencies, missing values, and variation in data quality. Nevertheless, the model was developed using routinely reported data to ensure its feasibility for application across a large number of farms in the routine monitoring process. Although this enhances practical applicability, it also means that model performance may be affected by limitations inherent in the underlying administrative data. In a small number of cases (2.6% of the dataset), the residential address required to calculate the distance between the farm and the farmer’s residence was unavailable. While most of these cases were attributable to farms without relevant production activity in the preceding period, some may have been linked to farm closures following animal welfare violations. Consequently, a limited degree of target leakage cannot be fully excluded for this predictor, although the affected observations represented only a small proportion of the dataset.

In addition, some variables, particularly antibiotic dispensing data, are subject to a substantial time lag, which may limit their temporal relevance for prediction.

Furthermore, the model relies on proxy variables for welfare, such as antibiotic use, mortality, and slaughterhouse findings, as no directly measured animal welfare indicators were available. While these variables provide meaningful insights into underlying farm management and animal health, they cannot fully capture the multifaceted nature of animal welfare issues on farm.

The model was compared with random selection as an operational benchmark for the ranking task. However, it was not directly compared with the existing Styrian selection procedure. A valid retrospective comparison would require reconstruction of the selection decisions under the existing procedure, including its multiple control purposes, risk-based components, administrative information and professional assessment. These elements could not be fully reconstructed from the available data. The relative effectiveness of model-based ranking and the existing selection procedure therefore remains unknown. In addition, no model-based comparator, such as penalized logistic regression, was evaluated. Such a comparison would require predefined decisions regarding the handling of missing values, categorical variables and potentially nonlinear relationships.

The predictive performance of the model was moderate but clearly above random selection, indicating that relevant patterns can be identified from the available data. However, performance may be limited by the relatively small number of farms with recorded violations.

### 4.9 Practical implication

From a practical perspective, the proposed model is intended to support veterinary authorities in prioritizing farms for official animal welfare inspections and to serve as a decision-support tool within a risk-based sampling approach. Rather than automatically triggering additional monitoring or inspections, the model could be used to identify farms with a higher predicted risk and thereby support a more targeted allocation of available inspection resources.

## 5 Conclusion

Despite these limitations, the results highlight the potential of routinely collected data to support risk-based prioritization of animal welfare controls using machine learning. The level of antibiotics dispensed for use on cattle farms, membership of the Animal Health Service and age of the farmer had the highest impact for prediction of animal welfare violations. In addition to antibiotic use, the results of meat inspections and data from fallen stock rendering plants also significantly influenced the model, although the results were somewhat heterogeneous.

Many factors associated with an increased risk of animal welfare violations may initially seem controversial. However, upon closer examination, they indirectly reveal insights into the general management practices of a farm and the farmers’ attitudes toward animal welfare. It is important to note that no one singular factor can be used to make decisions as to which farms require animal welfare inspections. Rather, the predictive model relies on the combined contribution of all included features, with each factor providing only part of the overall prediction. Further development of existing databases and the implementation of new data collection methods could enhance new pathways to collect data and therefore increase predictive performance. Applying this model to future animal welfare inspections will allow its real-world efficacy to be evaluated and determine whether it can support veterinary authorities in prioritizing farms for inspection and improve the allocation of inspection resources. The current model was developed for Styria, reflecting the state-level responsibility for inspection planning in Austria. In the future, a nationwide approach could be considered, although this would require appropriate legal adjustments, particularly regarding data protection and data exchange between federal states. The pooling of data from multiple federal states could increase the available dataset and the number of farms with documented welfare violations, potentially enabling more robust risk-based prioritization at the national level.

## Data Availability

The data used in this study were obtained from a national veterinary authority and are subject to data protection regulations. As a result, the data are not publicly available. Requests for access to the data should be directed to the respective authority and may be subject to approval. Requests to access these datasets should be directed to eva.obenaus@stmk.gv.at.
