# Supplementary material for: Risk-based prioritization of official animal welfare inspections using machine learning
Source: Front Vet Sci. 2026 Sep 30;13:1938469. doi: 10.3389/fvets.2026.1938469 (PMC13661325; doi:10.3389/fvets.2026.1938469)
Supplement: Supplementary file 1 [file Data_Sheet_1.docx]

***Supplementary Material***

# 1 Supplementary Data, Figures and Tables

**Supplementary Table S1.** Crosstable of number of farms with Deficiencies Catergory A-C classification and overall assessment 1-3*. Farms assigned to Category C or given an overall assessment 3 or 2 were classified as farms with animal welfare violations, colored in red.

|  | **Overall assessment: 3** | **Overall assessment: 2** | **Overall assessment: 1** |
| --- | --- | --- | --- |
| **Deficiencies Category C = criminal proceedings** | 20 | 45 | 12 |
| **Deficiencies Category B** | 9 | 55 | 154 |
| **Deficiencies Category A** | 11 | 83 | 198 |
| **No criminal proceedings** | 0 | 19 | 2516 |

* The A–C classification is assigned to the farm as a whole and ranges from A (deficiencies to be remedied within three months) to B (deficiencies requiring more than three months for correction) and C (immediate initiation of criminal proceedings). The 1–3 assessment is conducted separately for each animal species and relevant subcategory and represents a graded evaluation of the identified deficiencies and the required follow-up action, with category 3 also indicating that a criminal complaint should be initiated.

**Supplementary Table S2.** Hyperparameters search grid, and chosen values.

| **Hyperparameter** | **Search grid** | **Optimal value** |
| --- | --- | --- |
| Maximum number of boosting rounds/trees | 6000 | 6000 |
| Early-stopping rounds | 50 | 50 |
| Scale positive weight (spw) | 1, No-violation/violation ratio, sqrt of ratio | sqrt of ratio |
| Learning rate | 0.3, 0.2, 0.1, 0.05, 0.01, 0.005 | 0.005 |
| Maximum tree depth | 3, 5, 10, 15, 20, 25 | 15 |
| Minimum child weight | 1, 2, 3, 4, 5 | 2 |
| Row subsampling ratio | 0.7, 0.8, 0.9, 1 | 0.9 |
| Column subsampling ratio per tree | 0.7, 0.8, 0.9, 1 | 0.7 |
| Learning rate | 0.3, 0.2, 0.1, 0.05, 0.01, 0.005 | 0.005 |
| Minimum loss reduction required for a split (gamma) | 0, 1, 10, 100, 1000 | 0 |
| L2 regularization term (lambda) | 0, 0.01, 0.1, 1, 100, 1000, 10000 | 1 |
| L1 regularization term (alpha) | 0, 0.01, 0.1, 1, 100, 1000, 10000 | 0.01 |

**Supplementary Table S3.** train / test split of farms, and number of farms with specific set of inspections results.

| **Info** | **Value** |
| --- | --- |
| Number of unique farms | 2808 |
| Number of unique farms in train set | 2387 |
| Number of unique farms in test set | 421 |
| No. of farms with 1 inspection result | 2551 |
| No. of farms with 2 inspection results | 214 |
| No. of farms with 3 inspection results | 31 |
| No. of farms with 4 inspection results | 10 |
| No. of farms with 5 inspection results | 2 |

**Supplementary Table S4.** Descriptive statistic for all feature values.

| **Variable** | **mean** | **SD** | **Min** | **Max** | **Missing (%)** | **Is zero (%)** |
| --- | --- | --- | --- | --- | --- | --- |
| Single Application Form (CAP subsidies) | 0,84 | 0,37 | 0 | 1 | 0 | 16,24 |
| alpine cattle pasture | 0,34 | 0,47 | 0 | 1 | 48,27 | 34,14 |
| member of Animal Health Service | 0,54 | 0,5 | 0 | 1 | 2,18 | 45 |
| maximum age of farmer | 49,58 | 12,92 | 19 | 89 | 5 | 0 |
| number of managers | 1,17 | 0,41 | 1 | 5,00 | 0,00 | 0 |
| maximum distance between all farms of each associated person | 1527,7 | 8106,89 | 0 | 209719,00 | 2,18 | 81,17 |
| maximum number of farms of each associated person | 1,24 | 0,63 | 1 | 10,00 | 0,00 | 0 |
| maximum distance from farm to all people home address | 3503,79 | 16177,91 | 0 | 320442,00 | 2,18 | 7,01 |
| distance farm to farmers home address | 2583,26 | 15277,24 | 0 | 320442,00 | 2,66 | 8,2 |
| free range pig farming | 0,03 | 0,17 | 0 | 1,00 | 56,15 | 42,47 |
| production type turkey | 0,03 | 0,17 | 0 | 1 | 37,89 | 60,28 |
| production type chicken | 0,96 | 0,2 | 0 | 1 | 37,89 | 2,47 |
| production type undefined poultry | 0,04 | 0,19 | 0 | 1 | 37,89 | 59,74 |
| production type pullets (rearing) | 0,02 | 0,13 | 0 | 1 | 40,26 | 58,71 |
| production type poultry fattening | 0,08 | 0,27 | 0 | 1 | 40,26 | 54,96 |
| production type laying hens | 0,94 | 0,24 | 0 | 1 | 40,36 | 3,72 |
| production type free range poultry farming | 0,12 | 0,33 | 0 | 1 | 37,89 | 54,55 |
| production type organic poultry farming | 0,04 | 0,19 | 0 | 1 | 39,59 | 58,2 |
| livestock units cattle | 26,34 | 28,18 | 0 | 356,8 | 48,27 | 2,15 |
| livestock units poultry | 3,42 | 15,29 | 0 | 240 | 37,89 | 1,73 |
| livestock units horse | 5,28 | 11,72 | 0 | 198,8 | 83,28 | 1,41 |
| livestock units sheep | 5,86 | 10,82 | 0 | 103,7 | 77,93 | 2,69 |
| livestock units pigs | 31,44 | 60,06 | 0 | 1297,76 | 56,15 | 4,8 |
| livestock units goat | 1,19 | 2,13 | 0 | 18,32 | 86,13 | 2,53 |
| number of Animal Health Service veterinarians per district | 0,01 | 0,02 | 0 | 0,38 | 0 | 50,32 |
| total dispensed antibiotic cattle | 1,31 | 6,52 | 0 | 160,06 | 48,27 | 25,98 |
| dispensed antibiotics Category B cattle | 0,22 | 0,62 | 0 | 10,74 | 48,27 | 35,65 |
| dispensed antibiotics Category C cattle | 0,2 | 2,17 | 0 | 64,53 | 48,27 | 35,68 |
| dispensed antibiotics Category D cattle | 0,89 | 5,81 | 0 | 142,78 | 48,27 | 28,83 |
| total dispensed antibiotic pigs | 2,54 | 8,45 | 0 | 189,82 | 56,15 | 25,66 |
| dispensed antibiotics Category B pigs | 0,19 | 0,63 | 0 | 9,3 | 56,15 | 30,88 |
| dispensed antibiotics Category C pigs | 0,27 | 1,29 | 0 | 33,83 | 56,15 | 31,84 |
| dispensed antibiotics Category D pigs | 2,08 | 7,66 | 0 | 179,95 | 56,15 | 26,68 |
| no poultry production | 0,38 | 0,49 | 0 | 1 | 0 | 62,11 |
| relative number of C (animal welfare violation) poultry | 0,08 | 0,19 | 0 | 1 | 93,69 | 5,03 |
| relative number of D061 (Polyarthritis) poultry | 0 | 0 | 0 | 0,02 | 93,69 | 5,86 |
| relative number of D164 (multiple abscess) poultry | 0 | 0 | 0 | 0,02 | 93,69 | 2,66 |
| relative number of C (animal welfare violation) cattle | 0 | 0,01 | 0 | 0,25 | 55,12 | 44,27 |
| relative number of D061 (Polyarthritis) cattle | 0 | 0,01 | 0 | 0,17 | 55,12 | 44,81 |
| relative number of D164 (multiple abscess) cattle | 0 | 0,02 | 0 | 0,5 | 55,12 | 44,39 |
| relative number of D164a (other findings) cattle | 0 | 0,01 | 0 | 0,33 | 55,12 | 44,78 |
| relative number of E167 (Pneumonia) cattle | 0,05 | 0,13 | 0 | 1 | 55,12 | 32,9 |
| relative number of E168 (Pericarditis) cattle | 0,01 | 0,03 | 0 | 0,5 | 55,12 | 42,6 |
| relative number of E169 (Pleuritis) cattle | 0,07 | 0,14 | 0 | 1 | 55,12 | 31,23 |
| relative number of E169a (Peritonitis) cattle | 0 | 0,02 | 0 | 0,25 | 55,12 | 43,66 |
| relative number of E167 (Pneumonia) sheep | 0,15 | 0,23 | 0 | 1 | 92,18 | 4,58 |
| relative number of E169 (Pleuritis) sheep | 0,01 | 0,04 | 0 | 0,5 | 92,18 | 6,76 |
| relative number of D061 (Polyarthritis) pigs | 0 | 0 | 0 | 0,04 | 74,76 | 16,56 |
| relative number of D164 (multiple abscess) pigs | 0 | 0,01 | 0 | 0,08 | 74,76 | 14,13 |
| relative number of D164a (other findings) pigs | 0 | 0 | 0 | 0 | 74,76 | 24,86 |
| relative number of E167 (Pneumonia) pigs | 0,18 | 0,16 | 0 | 0,79 | 74,76 | 4,8 |
| relative number of E168 (Pericarditis) pigs | 0,02 | 0,03 | 0 | 0,23 | 74,76 | 6,92 |
| relative number of E169 (Pleuritis) pigs | 0,09 | 0,11 | 0 | 0,81 | 74,76 | 5,54 |
| relative number of E169a (Peritonitis) pigs | 0 | 0 | 0 | 0,05 | 74,76 | 19,31 |
| relative number of E169gs (adhesion/sticking of entrails) pigs | 0,01 | 0,03 | 0 | 0,25 | 74,76 | 11,82 |
| relative number of dead horses | 0,05 | 0,16 | 0 | 1 | 83,28 | 14,22 |
| relative number of dead sheep | 0,04 | 0,1 | 0 | 1 | 77,93 | 13,77 |
| relative number of dead goats | 0,03 | 0,14 | 0 | 1 | 86,13 | 11,98 |
| ratio dead animals to stock figure (in LU) | 0,1 | 2,38 | 0 | 84,97 | 37,89 | 60,7 |
| relative number of abortus (cattle) | 0,01 | 0,06 | 0 | 1 | 48,27 | 48,62 |
| relative number of dead calves (<6 months) | 0,05 | 0,11 | 0 | 1 | 48,27 | 34,72 |
| relative number of dead young cattle (6-24 months) | 0,01 | 0,03 | 0 | 0,67 | 48,27 | 44,94 |
| relative number of dead cattle (>24 months) | 0,02 | 0,05 | 0 | 1 | 48,27 | 38,15 |
| relative number of dead cattle unknown age | 0 | 0 | 0 | 0,12 | 48,27 | 50,99 |
| relative number of dead pigs | 0,05 | 0,62 | 0 | 22,33 | 56,15 | 23,03 |
| relative number of dead piglets | 0,02 | 0,15 | 0 | 3,17 | 60,95 | 37,25 |
| no cattle production | 0,48 | 0,5 | 0 | 1 | 0 | 51,73 |
| calf fattening | 0,04 | 0,2 | 0 | 1 | 48,27 | 49,65 |
| cattle fattening | 0,23 | 0,42 | 0 | 1 | 48,27 | 39,81 |
| dairy | 0,44 | 0,5 | 0 | 1 | 48,27 | 29,12 |
| suckler cow husbandry/fattening | 0,29 | 0,45 | 0 | 1 | 48,27 | 36,61 |
| no pig production | 0,56 | 0,5 | 0 | 1 | 0 | 43,85 |
| pig gilt rearing | 0,13 | 0,34 | 0 | 1 | 56,15 | 38,02 |
| mixed pig farming | 0,45 | 0,5 | 0 | 1 | 56,15 | 23,96 |
| pig fattening | 0,27 | 0,45 | 0 | 1 | 56,15 | 31,93 |
| no pig stock during evaluation period | 0,11 | 0,31 | 0 | 1 | 56,15 | 39,11 |
| pig breeding | 0,03 | 0,18 | 0 | 1 | 56,15 | 42,38 |
| no sheep production | 0,78 | 0,41 | 0 | 1 | 0 | 22,07 |
| no goat production | 0,86 | 0,35 | 0 | 1 | 0 | 13,87 |
| no horse production | 0,83 | 0,37 | 0 | 1 | 0 | 16,72 |

**Supplementary Table S5.** Explanation for missing values and zero values in the data set.

| **Variable** | **What missing means** | **What 0 means** |
| --- | --- | --- |
| alpine cattle pasture | not a cattle farm | is registered cattle farm, not registered Alpine pasturing |
| member of Animal Health Service | farm wasn't registered previous year | not a TGD member |
| maximum age of farmer | missing or incorrect birth date | / |
| distances farm/farmers | farm wasn't registered previous year, had incorrect or missing addresses, ... | rounded distance is 0 km |
| Livestock units_* | not a farm with this animal species | is registered for this animal species, no animals that year |
| total dispensed antibiotic_* | not a farm with this animal species | is registered for this animal species, no antibiotics for any group |
| dispensed antibiotics_#_* | not a farm with this animal species | is registered for this animal species, no antibiotics for this group |
| relative number of_+_* | not a farm with this animal species | is registered for this animal species, no findings for this SFU code |
| Relative number/ratio of dead_* | not a farm with this animal species | is registered for this animal species, no dead animals of this group |
| free range pig farming | not a farm with this animal species | is registered for this animal species, doesn't have animals of this group |
| production type poultry_* | not a poultry farm | is registered poultry farm and doesn’t have this poultry production type or animal type |
| free range poultry farming | not a poultry farm | is registered poultry farm, doesn't have free range poultry farming |
| organic poultry farming | not a poultry farm | is registered poultry farm, is not registered as Bio farm |
| production type cattle_* | not a farm with this animal species | is registered for this animal species, doesn't have animals of this group |

_*	Feature applies to all animal species (cattle, pigs, poultry, sheep, goats, and horses), including available species-specific subcategories (e.g. production type (diary, rearing, fattening, breeding, laying hens), age category (calves, cattle (6-24 or >24 months), animal type poultry (chicken, turkey, undefined))

_#	Features for the antibiotic category: B, C or D

_+	Features for the codes of the post-mortem and meat inspection for every specific animal species

**Sensitivity Analysis for a species-specific model:**
For the calculation of the general model 2.647 aggregated inspection results could be accounted for training. The species-specific models only have access to the animal welfare control data from farms with this particular species of animal (cattle, pigs, poultry, goats, sheep or horses). For testing the general model would have 475 rows. However, testing is filtered down according to the specific set of available data for each animal species. For each of them a comparison is made for prediction accuracy, including true positive (TP), true negative (TN), false positive (FP) and false negative (FN). The evaluation parameters include the AUC and MCC. Using the general model (Gen) resulted in better performance across nearly all evaluated metrics. The cattle-specific model was the only exception, showing slightly higher AUC and MCC values than the general model. Among the species-specific models (Spec.-sp.), the cattle model demonstrated the best overall performance. The general model showed a higher ability to correctly identify true positives, whereas the cattle-specific model identified more true negatives and generated fewer false positives. For all other species, the general model consistently outperformed the respective species-specific models. Although the cattle-specific model achieved slightly higher AUC and MCC values than the general model, the differences were relatively small. For pigs, poultry, small ruminants, and horses, the general model performed better than the corresponding species-specific models in terms of both AUC and MCC.

**Supplementary Table S6.** Comparison of performance metrics for species specific model to the general model

| **animals Species** | **Model** | **available training data** | **results for testing** | **TP** | **TN** | **FP** | **FN** | **AUC** | **MCC** |
| --- | --- | --- | --- | --- | --- | --- | --- | --- | --- |
| cattle | Gen | 2.647 | 214 | 24 | 83 | 105 | 2 | 0,71 | 0,24 |
|  | Spec-sp. | 1.270 | 214 | 12 | 158 | 35 | 9 | 0,73 | 0,28 |
| pigs | Gen | 2.647 | 175 | 11 | 122 | 38 | 4 | 0,74 | 0,31 |
|  | Spec-sp. | 839 | 175 | 8 | 98 | 63 | 6 | 0,63 | 0,10 |
| poultry | Gen | 2.647 | 246 | 6 | 165 | 74 | 1 | 0,73 | 0,19 |
|  | Spec-sp. | 1.321 | 246 | 2 | 96 | 148 | 0 | 0,54 | 0,07 |
| sheep and goats | Gen | 2.647 | 116 | 6 | 89 | 20 | 1 | 0,87 | 0,38 |
|  | Spec-sp. | 591 | 116 | 2 | 57 | 57 | 0 | 0,63 | 0,13 |
| horses | Gen | 2.647 | 59 | 5 | 37 | 16 | 1 | 0,73 | 0,34 |
|  | Spec-sp. | 348 | 59 | 4 | 21 | 34 | 0 | 0,59 | 0,20 |

Contrary to the assumption that a species-specific model provides better predictions by incorporating unique characteristics, the general model outperformed species-specific models in almost all performance metrics. By utilizing a broader dataset, the general model captured patterns that species-specific models may have missed due to limited sample sizes. This approach also reflects the structure of many farms, where multiple animal species are kept on the same holding and all species present are routinely assessed during official inspections. The general model exhibited higher true positive detection rates and generally produced better AUC and MCC scores, making it a more reliable model for identifying high-risk farms.

**Supplementary Table S7.** Antibiotic dispensing by TGD membership and animal species: number of farms and percentage of dispensing

| **TGD Membership** | **Animal species** | **No. of farms** | **total nDDDvet  > 0%** | **has nDDDvet D > 0 %** | **has nDDDvet C > 0 %** | **has nDDDvet B > 0 %** |
| --- | --- | --- | --- | --- | --- | --- |
| TRUE | pigs | 884 | 53.73 | 50.79 | 35.86 | 39.14 |
| TRUE | cattle | 951 | 72.13 | 65.4 | 47 | 48.05 |
| FALSE | pigs | 482 | 19.29 | 18.5 | 12.03 | 12.24 |
| FALSE | cattle | 664 | 17.77 | 14.01 | 8.13 | 6.78 |
| - | pigs | 3 | 0 | 0 | 0 | 0 |
| - | cattle | 0 | - | - | - | - |

Has nDDDvet > 0 counts percentage only out of those that do have that value available (is not missing - has production of that type).

## 1.1 Supplementary Figures

**Supplementary Figure S1.** Precision-recall curve of the general model.

**Supplementary Figure S2.** SHAP summary plot of features ranked 16–30. Features are ordered by decreasing mean absolute SHAP value. Each point represents a farm. SHAP, Shapley Additive Explanations.
\# SFU category E = pathological findings, but limited to certain organs, carcass not condemned
DDDvet = Defined Daily Dose for veterinary medicine
CAP = Common Agricultural Policy

**Supplementary Figure S3.** SHAP summary plot of features ranked 31–40. Features are ordered by decreasing mean absolute SHAP value. Each point represents a farm. SHAP, Shapley Additive Explanations.
\# SFU category E = pathological findings, but limited to certain organs, carcass not condemned
DDDvet = Defined Daily Dose for veterinary medicine
* SFU category D = carcass condemnation
